# Supplementary material for: Exposure to Disinfectants and Cleaning Products and Respiratory Health of Workers and Children in Daycares: The CRESPI Cohort Protocol
Source: Int J Environ Res Public Health. 2023 May 21;20(10):5903. doi: 10.3390/ijerph20105903 (PMC10218308; doi:10.3390/ijerph20105903)
Supplement: Supplementary file 1 [file ijerph-20-05903-s001.zip › ijerph-2286428-supplementary.pdf]

Figure S1. Various steps of the set-up of the CRESPI study

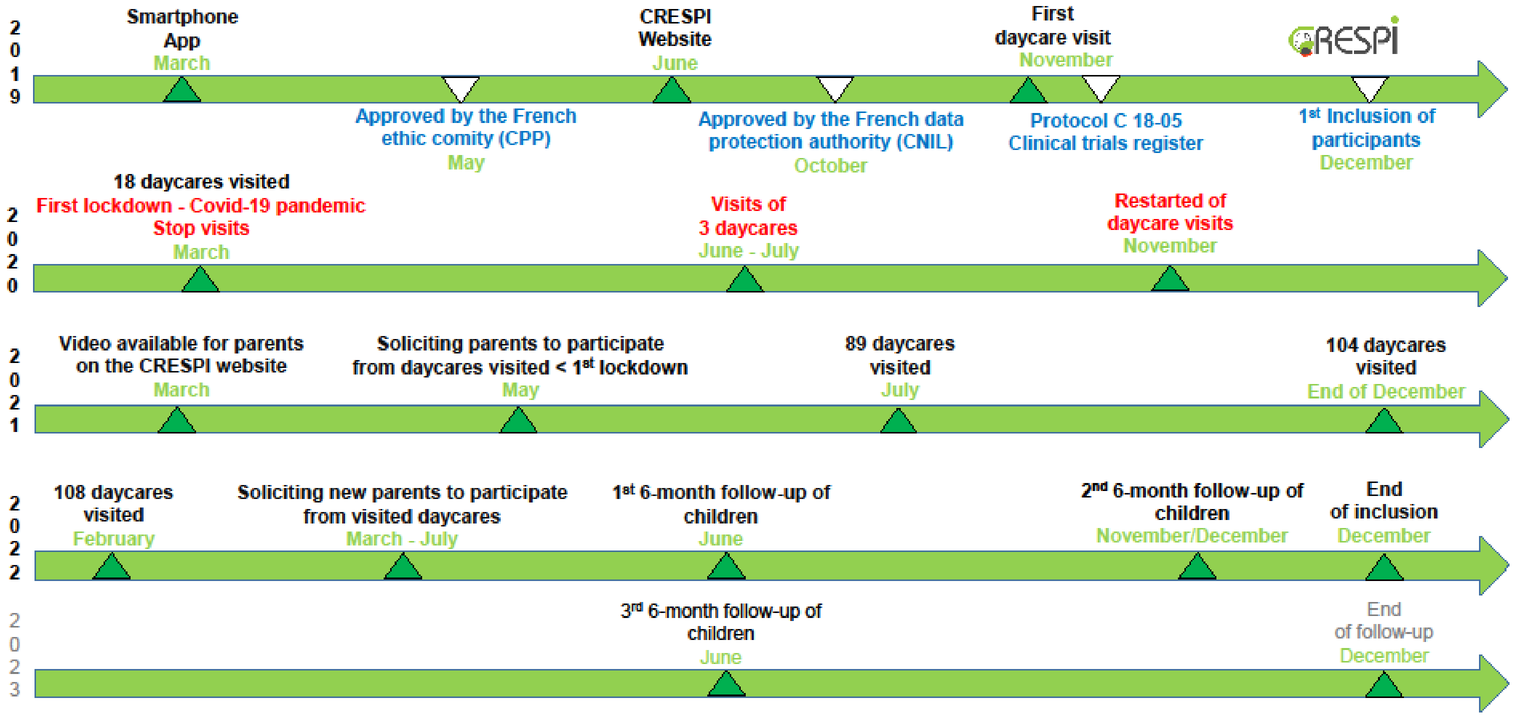

Figure S2. Databases available in the CRESPI cohort

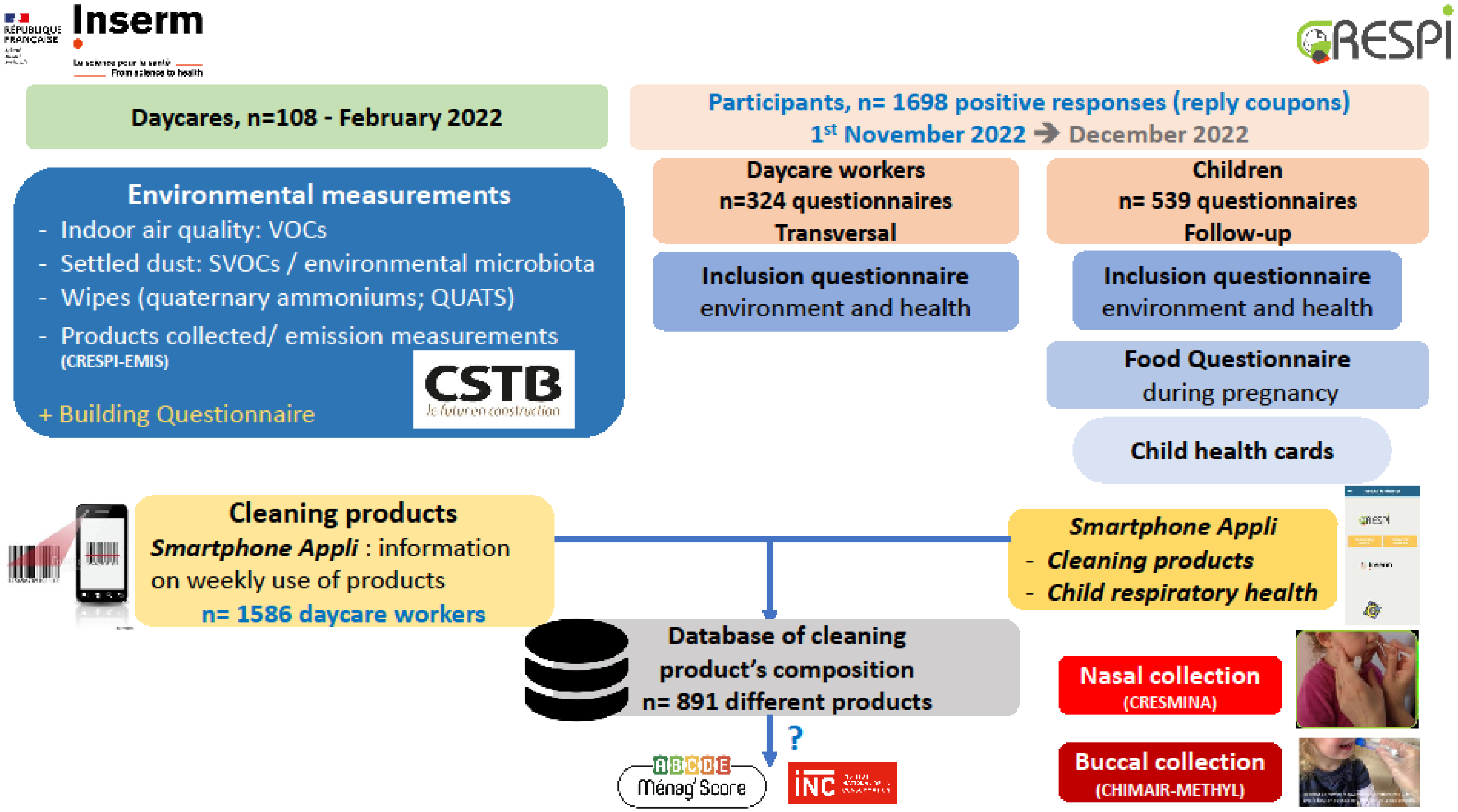

Table S1. Extract of the cleaning products' composition database

Barcode GENCOD	Product category	Form	Ingredient 1	Ingredient 2	Ingredient 3
1 017 496	Hand soap	Liquid	Water	Disodium lauroamphodiacetate	Sodium Lauryl Sulfate
7 270 178	Hand soap	Liquid	Water	Sodium Laureth Sulfate	Cocamidopropyl betaine
8 111 289	Multi-surface disinfectant	Spray	Water	Ethanol	Isopropanol
8 231 228	Dishwashing liquid	Liquid	Water	Calendula floral water	Lavender floral water
8 692 603	Hydroalcoholic gel	Gel	Ethanol	Water	Glycerin
